# Genomic Characterization of *Flavobacterium psychrophilum* Serotypes and Development of a Multiplex PCR-Based Serotyping Scheme

**DOI:** 10.3389/fmicb.2017.01752

**Published:** 2017-09-12

**Authors:** Tatiana Rochat, Erina Fujiwara-Nagata, Ségolène Calvez, Inger Dalsgaard, Lone Madsen, Alexandra Calteau, Aurélie Lunazzi, Pierre Nicolas, Tom Wiklund, Jean-François Bernardet, Eric Duchaud

**Affiliations:** ^1^Virologie et Immunologie Moléculaires, Institut National de la Recherche Agronomique, Université Paris-Saclay Jouy-en-Josas, France; ^2^Department of Fisheries, Kindai University Nara, Japan; ^3^Biologie, Epidémiologie et Analyse du Risque, Institut National de la Recherche Agronomique, Oniris Nantes, France; ^4^National Veterinary Institute, Technical University of Denmark Kongens Lyngby, Denmark; ^5^CEA/Genoscope/LABGeM, CNRS-UMR8030, Université d’Evry – Université Paris-Saclay Evry, France; ^6^Mathématiques et Informatique Appliquées du Génome à l’Environnement, Institut National de la Recherche Agronomique, Université Paris-Saclay Jouy-en-Josas, France; ^7^Laboratory of Aquatic Pathobiology, Environmental and Marine Biology, Faculty of Science and Engineering, Åbo Akademi University Turku, Finland

**Keywords:** *Flavobacterium psychrophilum*, salmonid aquaculture, fish-pathogenic bacteria, genomics, serotype, mPCR

## Abstract

*Flavobacterium psychrophilum* is a devastating bacterial pathogen of salmonids reared in freshwater worldwide. So far, serological diversity between isolates has been described but the underlying molecular factors remain unknown. By combining complete genome sequence analysis and the serotyping method proposed by Lorenzen and Olesen (1997) for a set of 34 strains, we identified key molecular determinants of the serotypes. This knowledge allowed us to develop a robust multiplex PCR-based serotyping scheme, which was applied to 244 bacterial isolates. The results revealed a striking association between PCR-serotype and fish host species and illustrate the use of this approach as a simple and cost-effective method for the determination of *F. psychrophilum* serogroups. PCR-based serotyping could be a useful tool in a range of applications such as disease surveillance, selection of salmonids for bacterial coldwater disease resistance and future vaccine formulation.

## Introduction

Salmonids represent one of the most important fish groups in the aquaculture industry (FAO, 2016). However, the success and sustainability of salmonid aquaculture largely depend on disease control.

*Flavobacterium psychrophilum*, a bacterium belonging to the family *Flavobacteriaceae*, is considered one of the most important bacterial pathogens in freshwater salmonid aquaculture worldwide (Nematollahi et al., 2003). The two main clinical forms are rainbow trout fry syndrome (RTFS) and bacterial cold-water disease (BCWD) (Cipriano and Holt, 2005) that can both result in considerable economic losses (Antaya, 2008; Starliper, 2011). All salmonid fish, especially coho salmon (*Oncorhynchus kisutch*) and rainbow trout (*Oncorhynchus mykiss*) are susceptible to RTFS and BCWD, as well as ayu (*Plecoglossus altivelis*), a fish related to salmonids. These infections are associated with skin ulcers, necrotic myositis, septicemia as well as exophthalmia (Nematollahi et al., 2003). *F. psychrophilum* has occasionally been detected in non-salmonid fish, samples of water, sediments and biofilms from rivers receiving outlet water from infected fish farms (Madetoja et al., 2002; Starliper, 2011). Despite extensive research on vaccine development (Plant et al., 2009; Gómez et al., 2014) and the recent marketing authorization of a vaccine in Chile, treatment still essentially relies on antibiotic administration (Schmidt et al., 2000). Diversity among *F. psychrophilum* isolates was reported at different levels, and a variety of typing schemes (e.g., biotypes, serotypes, and genotypes) was proposed accordingly. However, the correlation between these different schemes (Madsen and Dalsgaard, 2000; Madetoja et al., 2001; Ngo et al., 2017) and the impact of the different types on the onset and severity of the infection remain poorly understood.

Gram-negative bacteria have a complex set of surface polysaccharides that include the lipopolysaccharide (LPS) as well as capsular polysaccharides (CPS) or exopolysaccharides (EPS) (Raetz and Whitfield, 2002). These structures are thought to play important roles in the pathogenicity, immune escape, serum resistance, inflammation, adhesion and biofilm formation (Finlay and McFadden, 2006). Serotyping is based on the immunogenicity of various surface-exposed bacterial structures (e.g., LPS, capsular polysaccharides, flagella, peptidoglycan, etc.) biosynthesized by genes belonging to the accessory genome. This represents an advantage over many molecular typing methods (e.g., 16S rRNA sequencing, ribotyping, MLST) that rely on core genome genes, which are not phenotypically relevant determinants. LPS is one of the most pro-inflammatory compounds of Gram-negative bacteria typically consisting of three regions: a hydrophobic domain known as lipid A, a non-repeating “core” oligosaccharide, and a distal polysaccharide (or *O*-antigen). The *O*-antigen greatly varies between and within species, providing the main basis for many serotyping schemes. The structure of the *O*-polysaccharide of *F. psychrophilum* strain CSF259-93 has been solved and found to be an unbranched polymer of trisaccharide repeating units (MacLean et al., 2001). A 70 kb region (*FP1299* to *FP1234*) encompassing genes predicted to be involved in the biosynthesis, export, modification and polymerization of polysaccharides was previously identified in the genome of *F. psychrophilum* JIP 02/86 (Duchaud et al., 2007). Several substitutions of genes enclosed in this region were noticed by comparing the genomes of strains CSF259-93 and JIP 02/86 (Wiens et al., 2014).

Serological differences among *F. psychrophilum* isolates were noticed since early studies and different serotyping protocols were proposed, resulting in the description of a varying number of serotypes. Wakabayashi et al. (1994) described two distinct serotypes (O-1 and O-2) among a collection of Japanese isolates; a third serotype (O-3) was added in 1999 (Izumi and Wakabayashi, 1999) and a fourth one (O-4) in 2003 (Izumi et al., 2003). In Denmark, Lorenzen and Olesen (1997) proposed a serotyping scheme based on three serogroups (Fp, Th and Fp^T^). Using these schemes, some degree of association between serotypes and host fish species was observed. For example, strong relationship between host and O serotypes was reported, O-1, O-2, and O-3 corresponding to isolates infecting Coho salmon, ayu, and rainbow trout, respectively (Izumi and Wakabayashi, 1999). Moreover, the co-existence of genetically and serologically diverse isolates within individual farms was detected in Finland (Madetoja et al., 2001) and in the United Kingdom (Ngo et al., 2017). Finally, Mata et al. (2002) identified as much as seven different serovars (1–7) and also reported obvious links with host fish species. This serological diversity might have important consequences for the selection of appropriate candidate strain(s) for vaccine development (Gómez et al., 2014 and references therein), selective breeding of salmonids for increased disease resistance (Leeds et al., 2010), follow-up studies, epidemiological surveillance, disease control (Ngo et al., 2017), as well as for a better understanding of virulence and host resistance traits.

In the present study, we used the serotyping method proposed by Lorenzen and Olesen (1997) together with genome-wide association studies to identify key molecular determinants of serotypes. Accordingly, a multiplex PCR-based serotyping scheme (mPCR) was developed and successfully applied to a collection of 244 bacterial isolates.

## Materials and Methods

### Genome Comparisons

The complete or draft genome sequences used in this study are listed in **Supplementary Table S1**. Genome comparisons were performed using the web interface MicroScope (Vallenet et al., 2017), which allows graphic visualization enhanced by a synchronized representation of synteny groups^1^. Comparison of the gene content between strains was done by pairwise proteome similarity search using BlastP Bidirectional Best Hit and the MicroScope default parameters (i.e., >80% protein identity, >80% coverage). Analysis of gene organization was conducted using syntons (i.e., maximal set of orthologous gene pairs displaying a conserved organization) constructed with MicroScope default parameters (i.e., orthologous gene set having the same local organization in two strains were computed using BlastP Bidirectional Best Hit or having at least 30% identity on 80% of the shortest sequence and a genomic co-localization with allowed gap set to 5 genes). Evolutionary relationships between strains were analyzed using the DNA sequences of 1549 single-copy genes of the core genome identified by single-linkage clustering (*E*-value ≤ 1*e*-5 in blastp v2.2.26 pairwise proteome comparisons, alignment with amino-acid sequence identity ≥85% and length ≥70% of the shortest sequence). To mitigate the effect of recombination that introduces random and often large number of linked nucleotide differences per diversification event, a whole-genome MLST approach was adopted (Jolley and Maiden, 2010). Pairwise distances were computed as the fraction of genes where alleles differ between two strains and a tree was then obtained and drawn using the ‘nj’ and ‘plot.phylo’ functions of R package ‘ape’ (Popescu et al., 2012).

### Bacterial Isolates and Serological Tests

The *F. psychrophilum* isolates used in the present study are listed in **Supplementary Table S1**. The antisera were raised in rabbits against the serotypes Fp^T^ (= NCIMB 1947^T^), Fd (= DIFR 950106-1/1), and Th (= DK002 = 900406-1/3) (Dalsgaard and Madsen, 2000). The slide agglutination tests were performed using cross-absorbed antisera as previously described (Lorenzen and Olesen, 1997). Briefly, bacteria were grown on tryptone yeast extract salts broth (Holt et al., 1993) with 1.1% agar for 4 to 6 days. Bacterial colonies were suspended in sodium acetate buffer (0.05 M NaCO_2_CH_3_, 0.1 M NaC1, pH 7.5) and heated at 55°C for 10 to 15 min, and 15 mM sodium azide was subsequently added. Absorbed anti-Th, anti-Fd and anti-Fp^T^ antisera were used undiluted. Ten microliter of antiserum was allowed to react with 10 to 15 μL of the bacterial suspensions on a slide using a gentle rocking motion. The reaction was recorded macroscopically against a dark background after 1 to 2 min. Controls for autoagglutination were done in saline.

### Multiplex PCR

DNA was extracted using a Wizard genomic DNA purification kit (Promega) according to the manufacturer’s instructions. Primers (**Table 1**) were designed using Primer3 web version 4.0.0^2^ according to the complete and draft genome sequences available and conservation of their sequences was verified. *FP0711*, a core-genome gene highly conserved in *F. psychrophilum* was used as a positive control for DNA amplification. mPCR reactions were performed using DreamTaq DNA polymerase, 10× DreamTaq buffer (20 mM MgCl_2_) (Thermo fisher scientific) and 10 μM of each primer in a 50 μL final reaction volume. The mPCR amplification mix was heated at 95°C for 5 min, followed by 30 cycles of 95°C for 30 s, 52°C for 30 s, 72°C for 60 s, and a final extension at 72°C for 10 min. The amplified products were electrophoresed in 2.0% agarose gels run in 1× Tris–borate buffer with the GeneRuler DNA ladder mix (Thermo Fisher Scientific) as the molecular size standard.

**Table 1:** Oligonucleotides used in this study.

Name	Sequence	Amplicon size
ctrol_fw	AGCAAATTTGGCTCTTTTGG	188 bp
ctrol_rev	TTGTAACAACGCCACCAGTT	
Type-1_fw	ACCAACCTTCAAGATTATCGT	549 bp
Type-1_rev	GGGGAGTGGTTAGAACTGA	
Type-2_fw	TTGAACGAAACTTATATGGATAGA	841 bp
Type-2_rev	TTACCAAAGAGCCCTTTAGTG	
Type-3_fw	CGCCATGCAAGAAATTAGTT	361 bp
Type-3_rev	CCTGCGATCTCAACATATCA	

### Statistical Analysis

The putative association between genotypes and host fish species was investigated using chi-squared tests. *Post hoc* tests were conducted by comparing chi-squared residual values with normal distribution (the corresponding *p*-values were adjusted for multiple testing using the Bonferroni correction procedure). These analyses were conducted with the R package.

## Results

### Comparative Genomics Identify Key Molecular Determinants of the *F. psychrophilum* Serotypes

**FIGURE 1:**
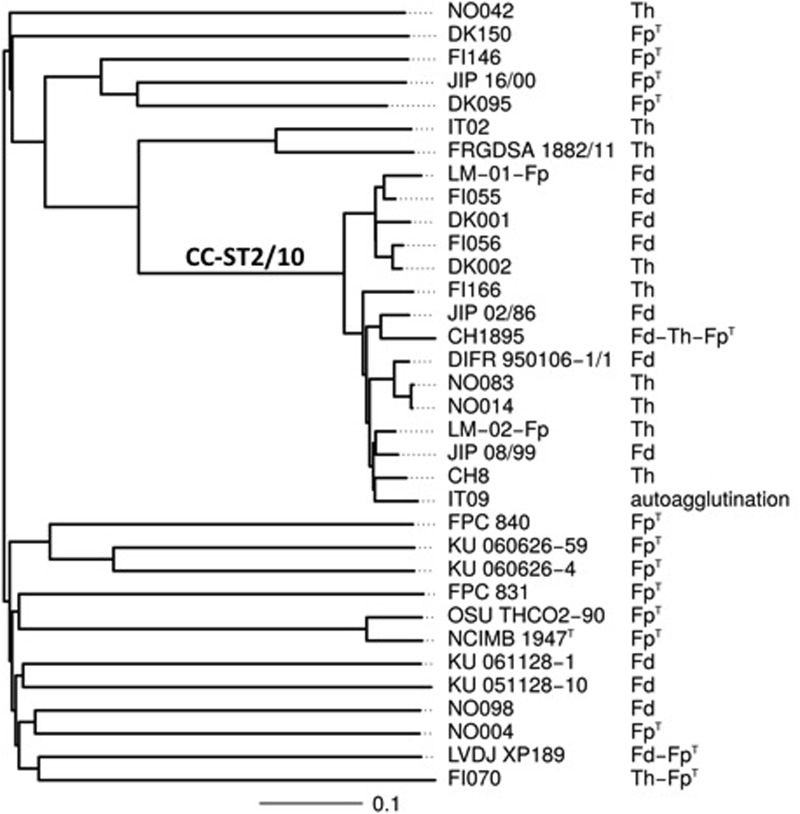
Serotypes and evolutionary relationships between the 34 *F. psychrophilum* strains. Serotypes are shown on a tree attempting to capture the phylogenetic relationships between the 34 *F. psychrophilum* strains used in this study for which complete genomes were available. The tree was constructed based on whole-genome MLST profiles (see Materials and Methods for details). Distances are expressed in terms of the fraction of genes where alleles differ between strains. The branch that encompasses strains belonging to clonal complex 2/10 is indicated as CC-ST2/10.

**FIGURE 2:**
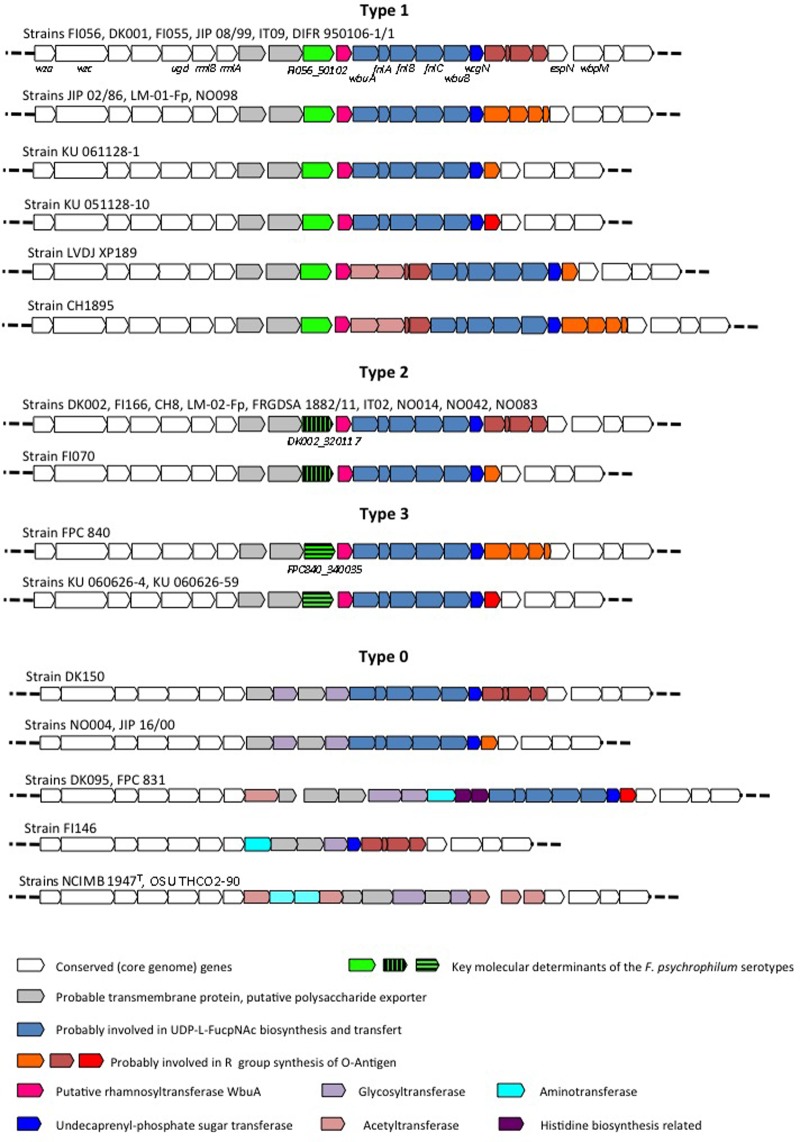
Genomic organization of the polysaccharide encoding loci. The polysaccharide encoding loci were analyzed using the Microscope platform. Gene conservation was predicted using both homology and synteny criteria. White arrows represent core genome genes (i.e., genes conserved in the whole genome dataset which deduced proteins display >80% identity over >80% coverage) and colored arrows represent genes belonging to the variable genome (i.e., genes not conserved in the whole genome dataset).

Using the slide agglutination test proposed by Lorenzen and Olesen (1997), serotyping was performed on the 34 strains which complete genomes were available in our laboratories. This serotyping scheme is able to discriminate 3 serotypes: Fp^T^, Th, and Fd, with some strains reacting with multiple antisera. The results are shown in **Table 2**. Strikingly, all strains but one belonging to CC-ST2/10 display the serotypes Fd or Th (**Figure 1**). However, the distribution of strains with these serotypes does not strictly follow the tentative phylogeny based on whole-genome MLST. For instance, strains FI056 and DK002, though tightly clustered in the phylogenetic tree, belong to serotypes Fd and Th, respectively. Comparison of the gene content of these two strains revealed that they share 2243 genes (i.e., the core genome genes) but contain only four and two strain-specific genes, respectively. Two of them (*FI056_50102* and *DK002_320117*) are co-localized, substituting each other, and lie in the predicted polysaccharide biosynthesis locus (**Figure 2**). *FI056_50102* and *DK002_320117* are orthologous genes of *FP1290* and *FPSM_02202*, both encoding hypothetical proteins in strains JIP02/86 and CSF259-93, respectively. These genes are adjacent to *wbuA* encoding a rhamnosyl transferase (Miyamoto et al., 2010), predicted to be involved in the *O*-antigen biosynthesis.

**Table 2:** Serotyping of *F. psychrophilum* isolates.

Strain	Serotype	mPCR Type
FI056	Fd	1
DIFR 950106-1/1^∗^	Fd	1
JIP 02/86	Fd	1
LM-01-Fp	Fd	1
NO098	Fd	1
DK001	Fd	1
FI055	Fd	1
JIP 08/99	Fd	1
KU 061128-01	Fd	1
KU 051128-10	Fd	1
LVDJ XP189	Fd-Fp^T^	1
CH1895	Fd-Th-Fp^T^	1
IT9	auto-agglutination	1
DK002^∗∗^	Th	2
FI166	Th	2
CH8	Th	2
LM-02-Fp	Th	2
FRGDSA 1882/11	Th	2
IT02	Th	2
NO042	Th	2
NO083	Th	2
NO014	Th	2
FI070	Th-Fp^T^	2
NCIMB 1947^T∗∗∗^	Fp^T^	0
OSU THCO2-90	Fp^T^	0
DK150	Fp^T^	0
NO04	Fp^T^	0
JIP16/00	Fp^T^	0
DK095	Fp^T^	0
FPC 831	Fp^T^	0
FI146	Fp^T^	0
FPC 840	Fp^T^	3
KU 060626-04	Fp^T^	3
KU 060626-59	Fp^T^	3

^∗^*Strain DIFR 950106-1/1 was used to produce the anti-Fd antiserum*.

^∗∗^*Strain DK002 = 900406-1/3 was used to produce the anti-Th antiserum*.

^∗∗∗^*Strain NCIMB 1947^T^ was used to produce the anti-Fp^*T*^ antiserum*.

Extensive comparative genomic analysis of this locus revealed a globally well-conserved structural backbone. By grouping the strains harboring identical or nearly identical neighboring gene organization using *FI056_50102/DK002_320117* as the seed, we detected another group of genomes containing a third gene, named *FPC840_340035* in strain FPC840, that substitutes *FI056_50102/DK002_320117* at the same chromosomal location (**Figure 2**). Based on the presence/absence of these three genes, four main genomic organizations, hereafter designated “Types,” were identified. Strains FI056, DK002, and FPC840 are the typical examples of Type-1, Type-2, and Type-3, respectively. Type-0 encompasses a number of strains that do not belong to any of Types 1, 2 and 3 (i.e., not containing any genes related to *FI056_50102, DK002_320117*, or *FPC840_340035*) and displays a less-conserved genomic structure and a much broader genomic diversity. The type strain of *F. psychrophilum*, NCIMB 1947^T^, belongs to this type.

Comparing the serotypes of the 34 *F. psychrophilum* strains to their Type revealed a remarkable correlation (**Table 2**). Strikingly, all strains (*n* = 10) that only reacted with the anti-Fd antiserum belong to Type-1, all strains (*n* = 9) that only reacted with the anti-Th antiserum belong to Type-2, and all strains (*n* = 11) that only reacted with the anti-Fp^T^ antiserum belong to Type-0 (8 strains) or Type-3 (3 strains). A search for genes consistently present (>80% identity, >80% coverage) in all strains belonging exclusively to serotype Fd (i.e., FI056, DIFR 950106-1/1, JIP 02/86, LM-01-Fp, NO098, DK001, FI055, JIP 08/99, KU 061128-1, and KU 051128-10) and absent from all strains belonging exclusively to serotype Th (i.e., DK002, FI166, CH8, LM-02-Fp, FRGDSA 1882/11, IT2, NO042, NO083, and NO014) revealed a unique gene (*FI056_50102* and its orthologs from the above-mentioned genomes, i.e., *IB65_06085, FP1290, LM01FP_150098, NO098_460117, DK001_50101, FI055_170072, JIP0899_1410015, KU06112801_120020*, and *KU05112810_320061*, respectively). Conversely, a search for genes consistently present (>80% identity, >80% coverage) in all strains belonging exclusively to serotype Th and absent in all strains belonging exclusively to serotype Fd revealed a unique gene (*DK002_320117* and its orthologs from the above-mentioned genomes, i.e., *FI166_350102, CH008_450117, LM02FP_380101, FRGDSA1882_70059, IT2_340117, NO042_670087, NO083_60118*, and *NO014_350089*, respectively).

The following particular cases were noticed: strain LVDJ XP189 reacted with the anti-Fd and anti-Fp^T^ antisera; strain CH1895 reacted with the anti-Fd, anti-Th and anti-Fp^T^ antisera and displayed technical variability between replicates; and strain IT9 was untypable due to auto-aggregation; these three strains belong to Type-1. Finally, strain FI070 reacted with the anti-Th and anti-Fp^T^ antisera and belongs to Type-2.

### Development of a Multiplex PCR

**FIGURE 3:**
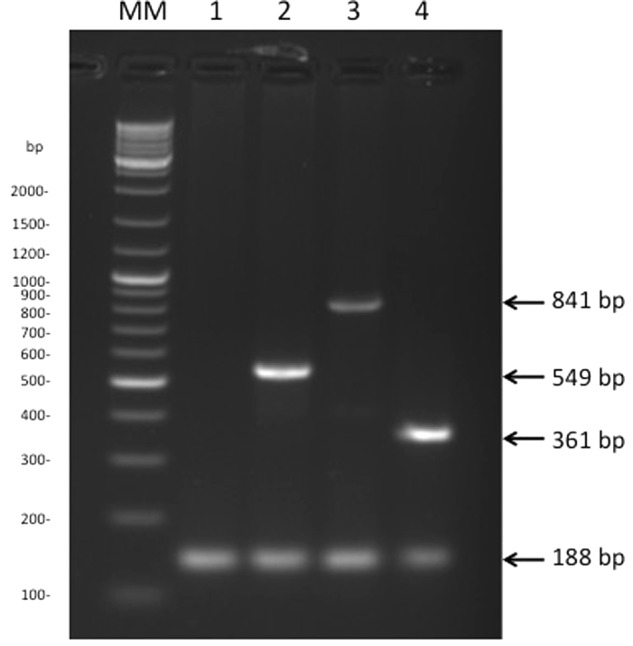
Multiplex PCR. Migration of the PCR products after Multiplex PCR amplification: (MM) GeneRuler^TM^ molecular marker; (1) strain NCIMB 1947^T^, revealing Type-0; (2) strain FI056 revealing Type-1; (3) strain DK002, revealing Type-2; (4) strain FPC 840, revealing Type-3.

Genes *FI056_50102, DK002_320117* and *FPC840_340035* do not display any primary sequence similarities. We therefore designed primers able to be used in a mPCR assay (**Table 1**). We included a positive control for PCR amplification that targets *FP0711*, a highly conserved gene ubiquitous in the *F. psychrophilum* genomes. To insure a straightforward discrimination of the types following gel electrophoresis, amplicons of 188, 361, 549 and 841 bp were selected for the positive control, Type-3, Type-1 and Type-2, respectively. Because of the diversity of Type-0 loci and the lack of a specific gene, no specific primers were designed for the latter and the amplification of only the control testified for Type-0 (**Figure 3**).

### Screening of a *F. psychrophilum* Strain Collection Using Multiplex PCR

The mPCR scheme was then used to screen a collection of 244 *F. psychrophilum* strains isolated from the fifties to 2015 from a wide variety of geographic areas and host fish species. All amplifications were successful and the results are show in **Supplementary Table S2**. This collection encompasses 28 strains that were included in the original paper of Lorenzen and Olesen (1997). A remarkable correlation between the previously reported serotypes obtained by these authors and the Types obtained using mPCR was observed for the 15 strains reacting with a unique antiserum using both agglutination and ELISA. Indeed: (i) the three strains (DIFR 911209-2, DIFR 930210-1, and DIFR 930413-1) that reacted exclusively with the anti-Fd antiserum belong to Type-1; (ii) the eleven strains (DIFR 900406-1, DIFR 910614-2, DIFR 910614-3, DIFR 910614-5, DIFR 911009-3, DIFR 911126-3, DIFR 930310-1, DIFR 930407-1, DIFR 930427-2, DIFR 930611-2, and Fi 147/93) that reacted exclusively with the anti-Th antiserum belong to Type-2; and (iii) the single strain (LFNW 16/90) that reacted exclusively with the anti-Fp^T^ antiserum belongs to Type-0. The situation of four cross-reacting isolates is more complex but in perfect agreement with Lorenzen and Olesen’s observations: i.e., Fd-Fp^T^ cross-reacting isolates using agglutination assay should be considered more closely related to Fd whereas Th-Fp^T^ cross-reacting isolates more closely related to Th. Using this interpretation, mPCR is consistent with the serotypes for four additional strains: (i) the three strains (LPAA 11522, Fi 171/93, and Fi 206/93) that reacted with both anti-Fd and anti-Fp^T^ antisera in agglutination assay and exclusively with the anti-Fd antiserum in ELISA assay belong to Type-1 and (ii) the strain (DIFR 910516-1) that reacted with both anti-Th and anti-Fp^T^ antisera in agglutination assay and exclusively with the anti-Th antiserum in ELISA assay belongs to Type-2. Finally, among the 28 isolates, the following particular cases (9 isolates) were noticed: (i) the two strains (LVDI 5/1 and Fi 196/93) that reacted exclusively with the anti-Fp^T^ antiserum in agglutination assay and that were untypable using ELISA belong to Type-1; (ii) the single strain (DIFR 910611-1) that reacted exclusively with the anti-Fp^T^ antiserum in both agglutination and ELISA belongs to Type-2; (iii) among the three strains that reacted with both anti-Fd and anti-Th antisera in agglutination but only with anti-Th in ELISA, DIFR 930324-1 belongs to Type-1 whereas Fi 88/93 and DIFR 910619-1 belong to Type-2; and finally (iv), three strains (USC PT 4.1, JIP 28/86 and LPAA 11524) that reacted with the anti-Fd, anti-Th and anti-Fp^T^ antisera in agglutination assay but only with the anti-Th antiserum in ELISA belong to Type-1. In their publication, Lorenzen and Olesen (1997) concluded than the ELISA assay discriminate different serotypes most distinctly than agglutination assay. If we compare ELISA results reported by these authors with mPCR results, 91% of correlation was found (22 out of the 26 ELISA-typable strains).

**FIGURE 4:**
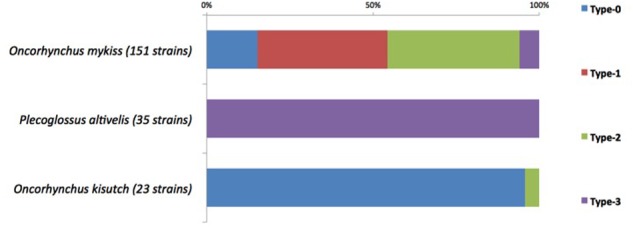
Association between Types and host fish species.

Our data clearly show a significant association between the Types and the host fish species (**Figure 4**): 22 out of the 23 strains retrieved from Coho salmon belong to Type-0 (*p*-value = 0), 59 and 60 out of the 151 strains retrieved from rainbow trout belong to Type-1 and to Type-2, respectively (*p*-values = 1.8*e*-7 and 5.8*e*-7) and all 35 strains retrieved from ayu belong to Type-3 (*p*-value = 0).

## Discussion

Serological differences among *F. psychrophilum* isolates were reported since the pioneering studies in the nineties and different serotyping schemes were proposed (Wakabayashi et al., 1994; Lorenzen and Olesen, 1997; Mata et al., 2002). However, the conventional protocols for the serotyping of *F. psychrophilum* isolates available so far suffer several drawbacks such as the choice of the appropriate scheme, the need to raise antisera from animals, and the bias of human interpretation of agglutination results. In addition, some strains display auto-agglutination, lack of reactivity, or cross-reaction with multiple antisera that make laborious reciprocal absorptions against heterologous strains necessary. Moreover, inconsistencies between the results of agglutination and ELISA have been reported (Lorenzen and Olesen, 1997). Hence, serotyping by conventional serological methods is costly, labor-intensive and requires significant technical expertise. Therefore, molecular serotyping using mPCR is a relevant alternative to traditional serotyping, though identification of molecular markers that determine the serotypes is a prerequisite in order to target them with DNA probes. Indeed, mPCR serotyping assays have been developed for a number of bacterial pathogens such as *Haemophilus parasuis, Pasteurella multocida, Salmonella* sp., *Streptococcus suis, S. pneumoniae* and *S. agalactiae* (Kong et al., 2005; Liu et al., 2013; Harper et al., 2015; Ma et al., 2016).

We took advantage of the availability of the whole-genome sequences of 34 *F. psychrophilum* isolates (Duchaud et al., in review) and of the previously published serotyping scheme of Lorenzen and Olesen (1997) to identify key molecular determinants of the serotypes. Focusing on strains FI056 and DK002 that are phylogenetically closely related but belong to two different serotypes (Fd and Th) (**Figure 1**), we identified the strain-specific genes *FI056_50102* and *DK002_320117* encompassed in the polysaccharide biosynthesis-encoding locus. Indeed, among the genes surrounding *FI056_50102* or *DK002_320117*, some are predicted to be involved in the biosynthesis of the nucleotide-sugar precursors of *O*-antigen (*rmlA* and *rmlB* for the synthesis of dTDP-L-rhamnose; *wbpM* for the synthesis of UDP-*N*-Acetyl-D-quinovosamine and *flnA*, *flnB* and *flnC* for the synthesis of UDP-*N*-Acetyl-L-fucosamine) whereas others encode the sugar transferases required for the assembly of lipid-linked O-repeat units (*wbuA*, *wbuB* and *wcgN*, the latter encoding the initial undecaprenyl-phosphate sugar transferase) (**Figure 2**). Previous studies aiming at identifying *F. psychrophilum* antigens recognized by polyclonal rabbit antisera have been performed (Crump et al., 2001, 2003). LPS-containing *O*-polysaccharide and core-region lipo-oligosaccharide have reported to be immunological relevant structures targeted by these sera. It is therefore tempting to speculate that the *O*-polysaccharide, predicted to greatly vary within species, provides the antigenic specificity of the serotyping scheme used in this study. So far, the *O*-polysaccharide structure has been solved for only one *F. psychrophilum* strain (CSF259-93), identified as belonging to Type 2 by mPCR (MacLean et al., 2001). Extensive biochemical analyses will be required for each *F. psychrophilum* serogroup to formally identify the nature of the carbohydrates targeted by each anti-serum.

Genes *FI056_50102*/*DK002_320117* were therefore promising candidate markers for serotype affiliation. Following a genome-wide association study using the 34 *F. psychrophilum* isolates, four Types were defined and Types 1 and 2 were firmly associated to Fp or Th serotypes (**Table 2**). Type 3 was not associated to an exclusive serotype in the scheme defined by Lorenzen and Olesen as Fp^T^ serotype was shared between Type 0 and Type 3 isolates. However, its strong association with ayu (**Figure 4** and **Supplementary Table S2**) indicates that Type 3 likely corresponds to the O2 serotype defined by Izumi and Wakabayashi (1999). Remarkably, neither the three genes located at this position (*FI056_50102, DK002_320117* and *FPC840_340035*) nor their encoded proteins display any primary sequence identity. Initially annotated as “hypothetical protein,” they are all predicted to be multipass transmembrane proteins, located in the inner membrane, with weak homologies to polysaccharide-biosynthesis proteins (e.g., *O*-antigen polymerase, *O*-antigen ligase) (**Table 3**). They likely perform the same general function (i.e., an unknown step in polysaccharide biosynthesis) but their activity should lead to structural variations such as the nature of the glycosidic bounds, additional sugar ramification(s), chain length, etc. As no obvious orthologous genes have been identified in strains belonging to Type 0, one might hypothesize that other multipass transmembrane proteins encompassed in polysaccharide biosynthesis-encoding locus could be functional homologs in these strains. Because of the phylogenetic incongruence of these genes, one must conclude that gene exchange by recombination took place at this locus. Indeed, *F. psychrophilum* was found to have a very high homologous recombination rate that contributes to population diversification (Nicolas et al., 2008; Vos and Didelot, 2009).

**Table 3:** *In silico* prediction of protein function.

Protein	BlastP (% identity; Eval)	COGnitor	InterProScan	HHpred on PfamA-30 (amino-acid positions; Eval)
FI056_50102	A7M6Y2 *O*-antigen polymerase Wzy of* Klebsiella pneumoniae* (22%; 0.0000000006)	COG3307 Lipid A core – *O*-antigen ligase and related enzymes	PF13425 *O*-antigen ligase-like membrane protein	PF13425 *O*-antigen ligase-like membrane protein (5 to 414; 8.7E-16)
DK002_320117	I6RSC6 Polysaccharide polymerase Wzy of *Streptococcus pneumoniae* (20%; 0.0000001)	No hit	No hit	PF14296 *O*-antigen polysaccharide polymerase Wzy (4 to 417; 1.8E-14)
FPC840_340035	I6RSC6 Polysaccharide polymerase Wzy of *Streptococcus pneumoniae* (23.27%; 0.00002)	No hit	No hit	PF01901 Putative *O*-antigen polymerase (24 to 415; 9.1E-14)

Taking advantage of this sequence divergence, we developed a mPCR assay able to discriminate strains belonging to Types 1, 2, 3 and 0. Our mPCR assay proved to be successful as all 244 strains tested were typable using this scheme (**Supplementary Table S2**). Among these strains, 28 were analyzed in the original paper of Lorenzen and Olesen (1997) and an outstanding correlation between the previously reported ELISA serotypes and the mPCR results was observed. The situation is more complex using agglutination assay. As a matter of fact, one should keep in mind that the antisera consist of polyclonal antibodies that were processed by multiple rounds of reciprocal absorptions against different heterologous strains. Therefore, cross-reactivity observed using slide-agglutination could be considered as a technical drawback, often noticed using such assays. Other variations in the locus (e.g., gene substitution, polymorphism or differences observed in other genes of the locus for some strains) may also impact the serotype and could explain the puzzling results that were obtained with the remaining cross-reacting isolates, especially since as much as seven different serotypes were reported for *F. psychrophilum* using a broader scheme (Mata et al., 2002). Using the latter scheme, strain FPC840 belongs to serovar 7 and using Izumi’s scheme it belongs to serovar O2. It is therefore tempting to speculate that Type-3 described in this paper actually corresponds to serovar 7 according to Mata and to serovar O2 according to Izumi. Another example is strain LVDI 5/1 that corresponds to serovar 5 using Mata’s scheme and to Fp^T^ using Lorenzen and Olesen’s (1997) scheme. An attempt to correlate all the previously published serotyping schemes with mPCR is proposed in **Supplementary Table S3**. Because Fp^T^ encompasses many strains displaying a poorer conservation of the locus together with a wider genomic diversity, one might suspect that they could also belong to some other serovars observed by Mata et al. (2002) or to other, yet undescribed serovars. One should conclude that the current serotyping schemes may not cover all existing serotypes of *F. psychrophilum* and that WGS is likely a promising and efficient approach to address this issue. Accordingly, our mPCR assay could be enriched by new targets allowing the detection of additional serogroups in the future.

Our results also revealed a striking association between mPCR-serotype and fish host species. All strains retrieved from coho salmon but one belong to Type-0. The only exception is the Chilean isolate MHC 1710K that belongs to Type 2. As previously proposed, Chilean fish farming practices have contributed to create overlapping host ranges as most fish farms often house mixed stocks of different salmonid species including coho salmon and rainbow trout (Avendaño-Herrera et al., 2009, 2014). The vast majority of strains retrieved from rainbow trout (119 among 151) belong to Types-1 and 2. However, 23 strains belong to Type-0 (20 from France, 2 from Switzerland, and one from Denmark) and 9 strains to Type-3 (8 from France and one from Israel). It is noteworthy that strains of serotypes Th and Fd were reported to be predominant in rainbow trout and associated to disease outbreaks (Lorenzen and Olesen, 1997; Dalsgaard and Madsen, 2000) and to tend to be more virulent than Fp^T^ isolates in experimental infection model using rainbow trout (Madsen and Dalsgaard, 2000; Madetoja et al., 2002). Considering that Types-1 and -2 correspond to Fd and Th serotypes, our results showed that Th and Fd are equally predominant (40%) in our rainbow trout set of isolates. Previous studies reported quite similar results, with serotype Th observed more frequently (60%) in the United Kingdom (Ngo et al., 2017) and Fd occurring more frequently in Finland (Madetoja et al., 2002). Altogether, the mPCR typing method represents a promising epidemiological tool as it allows determining if an isolate belongs to one of the two serotypes associated to disease outbreaks in European fish farms. Finally, all strains retrieved from ayu or their eggs belong to Type-3. It is therefore very tempting to speculate that the nature of the Type provides a selective advantage according to the host that is infected. Nevertheless, these associations might also reflect the underlying genetic structure of the population without necessarily involving a selective advantage linked to the serotype. Indeed, MLST analysis of *F. psychrophilum* population structure has also identified clear associations between particular clonal complexes and some fish host (Nicolas et al., 2008). Our identification of the genetic determinants of the serotypes provides a basis for future studies aiming at clarifying the links between serotypes, population structure, and virulence.

As previously observed for other bacterial pathogens, mPCR-based serotyping methods often perform better than traditional serotyping in terms of detection rate, accuracy, reproducibility and compatibility of the results between different laboratories (Harper et al., 2015; Ma et al., 2016). Therefore, our mPCR assay may be readily used in large-scale studies aiming at fish farming improvement. Indeed, this straightforward mPCR-based serotyping procedure might be very useful for a variety of non-academic purposes such as bacterial strain selection for vaccine development, selective breeding or epidemiological surveillance in fish farms.

## Author Contributions

TR: Substantial intellectual contribution throughout the study, data interpretation and manuscript preparation. EF-N, SC, and AL: Bacterial isolates management, DNA extraction and mPCR. ID and LM: Bacterial isolates management and serological tests. AC and PN: Participation in genomic data analysis and handling. TW and J-FB: Participation in data interpretation. ED: Substantial intellectual contribution throughout the study, gene mining, interpretation of data, manuscript preparation and responsible for acquisition of funding. All authors read and approved the final manuscript.

## Conflict of Interest Statement

The authors declare that the research was conducted in the absence of any commercial or financial relationships that could be construed as a potential conflict of interest.

## Footnotes

1 http://www.genoscope.cns.fr/agc/mage/

2 http://primer3.ut.ee

## Supplementary Material

TABLE S1 Genome sequences used in this study.

TABLE S2 Screening of the *Flavobacterium psychrophilum* strain collection using mPCR.

TABLE S3 Comparison of previously published serotyping schemes with mPCR.
